# Simulating and stimulating performance: introducing distributed simulation to enhance musical learning and performance

**DOI:** 10.3389/fpsyg.2014.00025

**Published:** 2014-02-04

**Authors:** Aaron Williamon, Lisa Aufegger, Hubert Eiholzer

**Affiliations:** ^1^Centre for Performance Science, Royal College of MusicLondon, UK; ^2^Department of Research and Development, Conservatory of Southern SwitzerlandLugano, Switzerland

**Keywords:** music education, distributed simulation, performance anxiety, performance science, virtual reality

## Abstract

Musicians typically rehearse far away from their audiences and in practice rooms that differ significantly from the concert venues in which they aspire to perform. Due to the high costs and inaccessibility of such venues, much current international music training lacks repeated exposure to realistic performance situations, with students learning all too late (or not at all) how to manage performance stress and the demands of their audiences. Virtual environments have been shown to be an effective training tool in the fields of medicine and sport, offering practitioners access to real-life performance scenarios but with lower risk of negative evaluation and outcomes. The aim of this research was to design and test the efficacy of simulated performance environments in which conditions of “real” performance could be recreated. Advanced violin students (*n* = 11) were recruited to perform in two simulations: a solo recital with a small virtual audience and an audition situation with three “expert” virtual judges. Each simulation contained back-stage and on-stage areas, life-sized interactive virtual observers, and pre- and post-performance protocols designed to match those found at leading international performance venues. Participants completed a questionnaire on their experiences of using the simulations. Results show that both simulated environments offered realistic experience of performance contexts and were rated particularly useful for developing performance skills. For a subset of 7 violinists, state anxiety and electrocardiographic data were collected during the simulated audition and an actual audition with real judges. Results display comparable levels of reported state anxiety and patterns of heart rate variability in both situations, suggesting that responses to the simulated audition closely approximate those of a real audition. The findings are discussed in relation to their implications, both generalizable and individual-specific, for performance training.

## Introduction

Exceptional musical performances require an ability to execute complex physical and mental skills on stage under intense pressure and public scrutiny. While many of these skills can be honed through deliberate practice (Ericsson et al., 1993), opportunities to gather experience on stage, which is simultaneously rich in contextual complexity and yet safe to allow musicians to experiment and develop artistically, are rare. Here, we report a new educational and training initiative aimed at creating realistic, interactive performance scenarios using simulation.

A high quality virtual environment should offer a three dimensional visual experience of weight, height, and depth and provide “an interactive experience visually in full real-time motion with sounds and possibly with tactile and other forms of feedback” (Roy, 2003, p. 177). The more elaborate the quality of the simulation, the more the feeling of immersion and perception of “reality” that is experienced by users. This also depends on the level of interactivity, dimensionality, accuracy, fidelity, and sensory input and output (Satava, 1993). When these features are addressed, simulation can be used as a performance tool to explore and study specific behaviors and as an educational tool to acquire and practice skills (Gallagher et al., 2005; Axelrod, 2007).

Thus far, successful application of virtual training environments has been shown in studies addressing fears of heights, flying, spiders, and public speaking. Slater et al. (2006), for instance, showed significant increased signs of anxiety in people with phobias when speaking in front of a neutrally behaving virtual audience. By comparing somatic and cognitive features, including the Personal Report of Confidence as a Speaker (PRCS) as well as heart rate measurements before, during, and after the performance, results suggest that such exposure can be an effective tool for treatment. A follow-up study by Pertaub et al. (2006) employed virtual audiences who could respond neutrally, positively, and negatively. Their verbal responses included expressions such as “I see” or “That's interesting,” moving to more evaluative statements like “That's absolute nonsense.” The audience was also able to provide non-verbal cues such as shifts in facial expression, changes in posture, and short animations including yawning, turning their heads, or walking out of the room. The results of comparing subjective measurements (e.g., PRCS) before and after virtual exposure showed that the way the audience responded directly affected participants' confidence as public speakers, in that positive audience reactions elicited higher confidence levels while the negative response caused a significant reverse effect.

Virtual environments have also been shown to be an effective tool for training elite performers such as pilots, athletes, and surgeons, especially when they have relatively little exposure to real-world performance contexts or when failure carries career- and/or life-threatening risks. In surgery, for example, such training, compared with more traditional methods, has been shown to reduce the number of errors in surgical procedures, enhance the rate and extent of skill acquisition, and improve planning strategies (e.g., Grantcharov et al., 2004; Xia et al., 2011). It not only helps trainees who lack experience in real-world surgical contexts but also advanced surgeons who require exposure to new procedures and technologies (Sutherland et al., 2006).

To a limited extent, virtual environments have been employed in music, where studies have tested their use in managing music performance anxiety. To date, the effects of exposure in these settings on psychological and physiological responses to performance stress are mixed. On the one hand, exposure to performing conditions using simulation has been shown to decrease state anxiety significantly compared with a control group, particularly for those musicians who score higher on *trait* anxiety measures (Bissonette et al., 2011). On the other, Orman (2003, 2004) found no discernible, consistent patterns of change in either self-reported anxiety levels or heart rate for musicians taking part in an intervention offering graded exposure to stressful performance situations. Such inconsistent results can also be found in other domains, for instance in studies examining surgical performance by Munz et al. (2004) and Torkington et al. (2001), who compared simulated training against more conventional training and no training at all. This emphasizes the importance of the backgrounds of individual participants, their levels of immersion (i.e., how realistically they experience the environment), and the quality of the interface on the overall effectiveness and range of uses of a virtual environment.

In terms of the studies conducted in music, it is possible that the inconsistent findings are due to differences in the fidelity and interactivity of the simulations used, but in addition, several methodological limitations should be highlighted in the extant research. Firstly, participants' use of anxiolytic medication and other substances that may have affected their perceptions of or physiological response to stress was not controlled. Secondly, while heart rate was measured in all studies, there were limitations with how the data were reported (Orman, 2003, 2004), or the results were not reported at all (Bissonette et al., 2011). Concerning the former, stressful events have been shown to influence temporal fluctuations of the peak-to-peak times in the electrocardiogram (ECG), the R-to-R (RR) interval, known as heart rate variability (HRV) (Berntson and Cacioppo, 2004). While the simplest measures of HRV are the mean of the RR time series and the standard deviation about its mean, both of these statistics are based on the *absolute* magnitude of the RR interval. However, in many applications, *relative* measures such as the power ratio of the low- and high-frequency components of HRV allow for more reliable comparisons between participants (for further information, see “Data treatment and analysis” and Williamon et al., 2013). Finally, the existing studies test the hypothesis that performance exposure using virtual environments can ameliorate psychological and physiological symptoms of performance anxiety; while this seems plausible on the surface, the inter- and intra-individual variability in how people experience and interpret such symptoms can be extremely large (Williamon, 2004). Given the multifaceted nature and impact of performance anxiety on musicians and the personal significance it holds even for highly experienced performers (Kenny, 2011), mere exposure to performance situations can be seen as the first of many steps in identifying and managing pernicious anxiety-related problems. Rather, it is possible that anxiety management will be only one of many possible uses of simulation training for enhancing musicians' learning and performance.

The aim of our research was to design, test, and explore possible uses of new interactive, simulated environments that provide salient cues from real-life performance situations—in this case, a recital and an audition. The environments were designed on the principles of “distributed simulation,” in which only a *selective abstraction* of environmental features are provided. Distributed simulation has been tested and applied widely in the field of surgical education, where fully-immersive virtual operating theatres are expensive to build, maintain, and run. The findings suggest that, indeed, a simulated environment only requires few environmental cues above and beyond the interactive simulation of an injury, wound, etc. in order to produce significant advancements in learning surgical techniques compared with more common training methods (Kassab et al., 2011). For instance, these may include a scaled-down operating lamp, background sounds played through loud speakers, and even life-sized pictures of machines commonly found in operating theatres; as the surgeons themselves do not operate such equipment and must focus on performing the procedure at hand, the imitated (yet plausible) environmental features often go unnoticed while adding significantly to the level of immersion experienced by participants. This approach has resulted in low-cost, convincing simulations that are portable, widely accessible, and can be used in almost any available space. In our study, advanced violin students performed the same piece in performance settings that employed a selective abstraction of recital and audition environments. The musicians' perceptions and experience of performing in the environments were obtained through questionnaires, and for a subset of participants, ECG data were recorded during their performances.

## Materials and methods

### Participants

Eleven violinists (6 men, 5 women; mean age = 22.45 years, *SD* = 2.25) were recruited for the study through the Royal College of Music's (RCM) student email list. They had been playing the violin for 16.18 years (*SD* = 2.75), including their first performance at age 6.50 (*SD* = 2.20). All students performed regularly (*M* = 2.32 times per month, *SD* = 1.30) and practiced on average for 3.93 h per day (*SD* = 0.85).

A subset of 7 participants from the larger sample (4 men, 3 women; mean age = 22.57 years, *SD* = 2.50) were selected based on their availability to take part in an additional performance for a real audition panel. These musicians had been playing for 16.00 years (*SD* = 3.21), with their first performance at age 7.57 (*SD* = 2.14). They performed for audiences on average 2.00 times per month (*SD* = 1.15) and practiced for 3.89 h per day (*SD* = 0.93).

This study was granted ethical approval by the Conservatoires UK Research Ethics Committee and was conducted according to ethical guidelines of the British Psychological Society. Informed consent was obtained from all participants, and no payment was given in exchange for participation.

### The performance simulator

**Figure 1:**
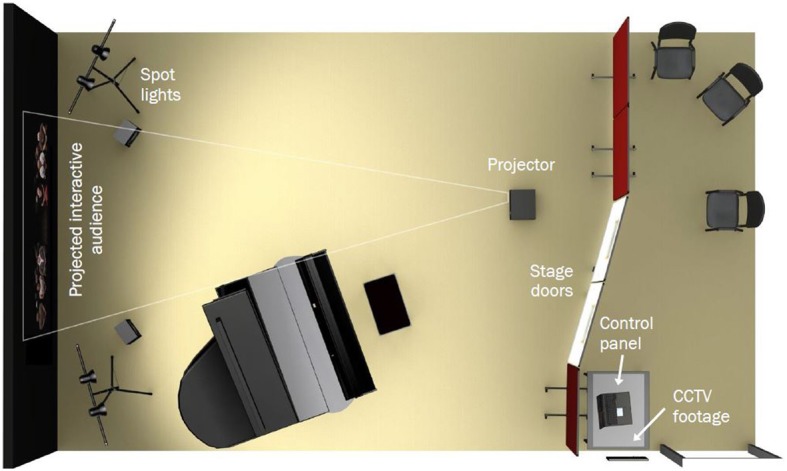
**The performance simulator, showing backstage (right) and stage (left) areas.** Backstage, CCTV footage of the virtual audience or audition panel is shown on a wall-mounted flat-screen monitor, and controls for operating the audience and audition panel are located on a nearby computer. On stage, a ceiling-mounted beamer projects the life-sized audience or audition panel onto a wall, with spot-lights and loudspeakers on both sides. Stage curtains (not shown) frame the projected image.

The performance simulator was developed through a collaboration of the first and third authors and London-based design consultancy Studiohead. The aim was to generate back-stage and on-stage environments using a selective abstraction of key features consistent across a wide range of Western classical performance venues. The selection of these features was informed by interviews with advanced musicians on their experiences and perceptions of performing (see Clark et al., in press) and on further pilot interviewing focused on performance environments. By undertaking user research, interviewing staff and students from the RCM and watching performances from backstage, common features could be identified and then recreated, including interaction with a backstage manager, the ritual of walking on stage, using appropriate lighting and sound cues (see Figure 1), and providing realistic, interactive virtual audiences of different types and sizes.

With these features in mind, the simulator was designed along the principles of distributed simulation (i.e., low-cost and portable, with high fidelity) to operate in two modes:

1. a recital with 24 virtual audience members
2. an audition situation with three “expert” virtual judges

In both simulations, pre- and post-performance protocols were employed that matched those found at leading international performance venues—for instance, entrance to a “green room” for warm-up, stage calls at regular intervals, and scripted procedures for entering, bowing, and exiting the stage (see “Procedure”). A short introduction to the simulator is provided in Movie 1 (see **Supplementary Material**).

#### Recital simulation

**Figure 2:**
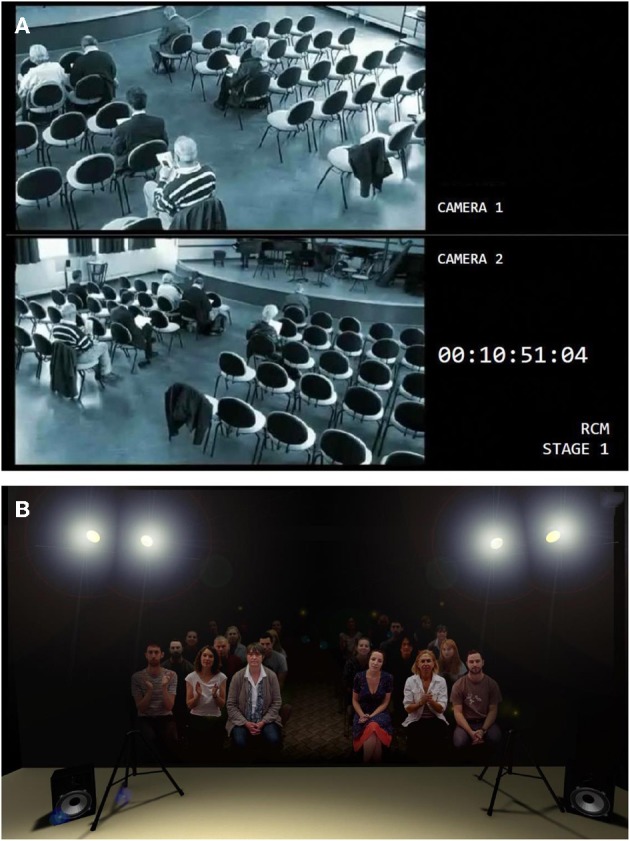
**(A)** A still from the CCTV footage displayed in the backstage area of the recital simulation. **(B)** A still of the virtual audience projected in the stage area, framed by spot-lights, loudspeakers, and (not shown) stage curtains.

To create an interactive audience and capture plausible audience behaviors, 11 concert-goers were filmed individually using green-screen technology sitting still while listening to a Western classical performance (with naturalistic body swaying, fidgeting movements, and coughing) and responding to a successful or unsuccessful performance with a standing ovation, enthusiastic applause, polite applause, or aggressive booing and displays of displeasure. They performed these tasks on the same approximate timeline, achieved by having them watch a video of an actor mounted next to the video camera and asking them to synchronize their behaviors with those of the actor. They were filmed individually so that audiences of different sizes could be compiled. For the purposes of this study, the audience consisted of 24 people seated in two blocks of chairs situated in a small auditorium designed using Adobe After Effects (CS5.5) (Figure 2). A Flash interface was created to enable the audience to be manipulated using pre-set control commands from a computer console located in the backstage area.

In terms of environmental cues, the backstage area was equipped with CCTV footage of audience members taking their seats in an auditorium (Figure 2). On stage, there were spot-lights and curtains on both sides of the projected audience. Noise distractions such as coughing, sneezing, and phone ringing were included in the stage area played through loudspeakers. Recordings of these, as well as all applause and booing, were made separately by another group of 16 volunteers in an anechoic chamber. Small recital hall reverberation was added to these recordings and then synchronized and layered on top of the video footage.

#### Audition simulation

**Figure 3:**
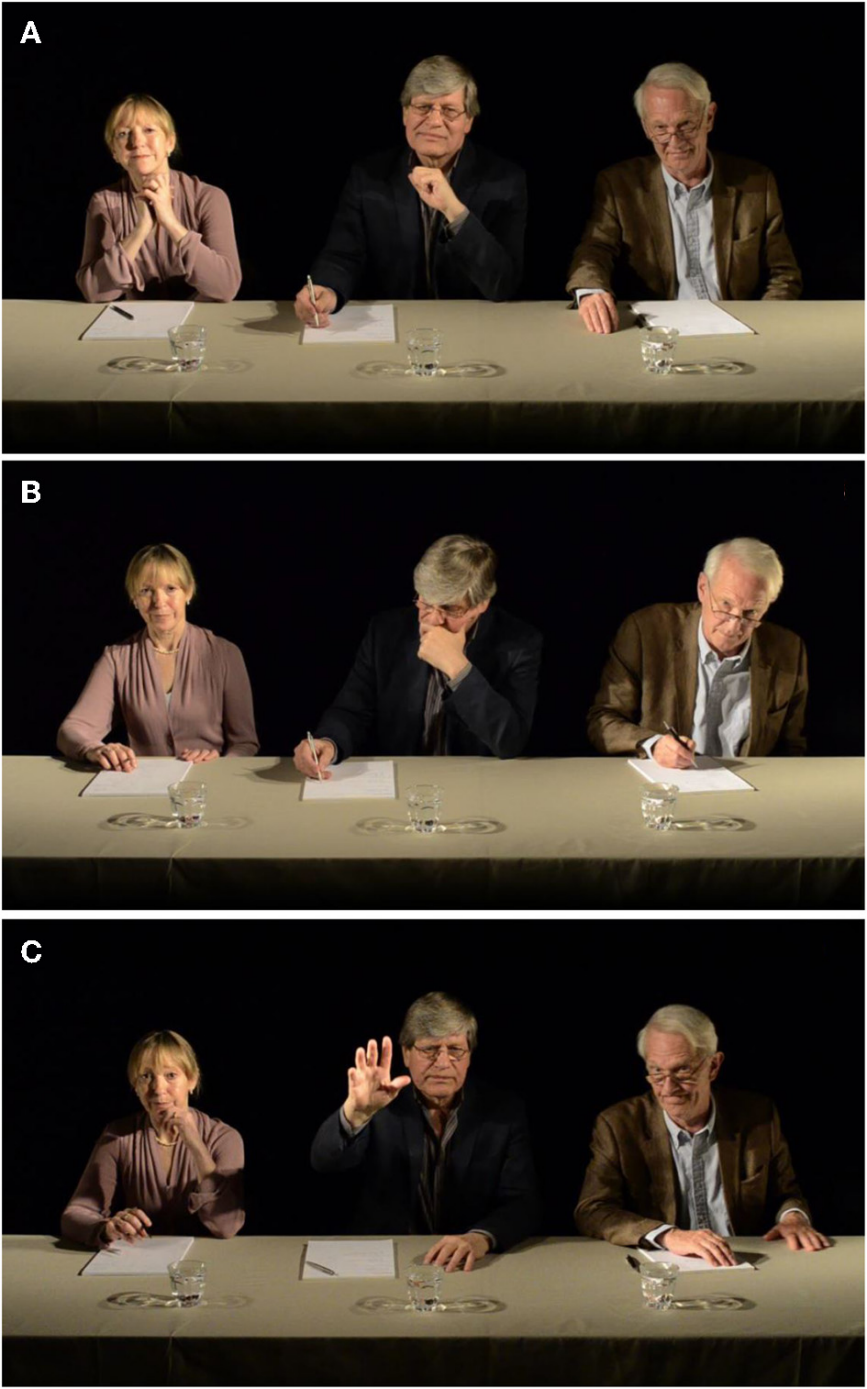
Stills of the virtual audition panel **(A)** responding positively, **(B)** showing displeasure, and **(C)** stopping the audition.

The members of the simulated audition panel were three professional actors who were filmed together sitting behind a table. Starting each audition with a neutral “Hello, please start whenever you are ready,” the panel showed typical evaluative behaviors such as making notes, looking pensively, and leaning back while simultaneously portraying positive, neutral, or negative facial expressions and behavioral feedback during the performance (e.g., smiling or frowning; leaning in toward the performer, or folding arms and leaning back). Each mode of listening was presented in loops of 5 min; the research team could change the mode of response after the loop or immediately. At the end of the performance, the audition panel could respond to the performance in a positive, neutral, or negative fashion—for instance, an enthusiastic “Thank you, that was excellent,” a polite but non-committal “Thank you very much,” a disappointed sigh followed by “Thank you for coming,” or a disruptive “Thank you, I think we've heard enough” displaying displeasure and frustration. To enhance the level of interactivity between the panel members, the actors were filmed together in a small room, and then a virtual audition room with black background was added using Adobe After Effects (CS5.5) (Figure 3). Similar to the recital simulation, a Flash interface was created to enable the panel to be manipulated using pre-set control commands from a computer console located in the backstage area.

The environmental cues in the audition simulation included CCTV footage displayed backstage of the panel chatting among themselves in hushed voices. On stage, there were spot-lights, loudspeakers, and curtains on both sides of the projected panel.

### Measures

#### Simulation evaluation questionnaire

A simulation evaluation questionnaire was based closely on work in surgical education by Kassab et al. (2011). It consisted of 19 statements about (1) general perceptions and experience of performing in the simulator, (2) the quality of the backstage experience, (3) the quality of the on-stage experience, and (4) the potential for using the simulator to develop performance skills. Each statement was rated on a 5-point Likert-type scale from 1 = “strongly disagree” to 5 = “strongly agree.” Table 1 shows all 19 statements, as well as descriptive statistics (mean, median, standard deviation) for each.

**Table 1:** **Descriptive statistics for questions in the Simulation Evaluation Questionnaire completed after performing in the recital and audition simulations**.

***Question***	***Recital***				***Audition***			
	***Median***	***Mean***	***SD***	***p***	***Median***	***Mean***	***SD***	***p***
***GENERAL PERCEPTIONS AND EXPERIENCE***								
1. The simulation (including backstage, the audience, spot-lights, etc.) provided a realistic experience	4.0	3.42	1.08	ns	4.0	3.67	0.50	0.005
2. The steps involved in the simulation (i.e., waiting backstage, walking on stage, etc.) closely approximated a real performance situation	4.0	3.75	0.75	0.013	3.5	3.50	1.00	ns
3. I behaved and presented myself in the same way as I do in a real performance	4.0	3.42	1.24	ns	4.0	3.67	0.98	0.046
***BACKSTAGE***								
4. The interaction with the backstage manager was realistic	4.5	4.00	1.20	0.018	4.0	3.92	1.24	0.029
5. The CCTV footage in the backstage area was realistic	3.0	2.83	1.40	ns	4.0	3.58	1.56	ns
6. The sounds heard in the backstage area were realistic	3.5	3.42	1.08	ns	4.0	4.00	1.12	0.028
7. The decor of the backstage area (including signage and lighting) was realistic	4.0	3.67	0.49	0.005	3.5	3.42	1.08	ns
***ON STAGE***								
8. The transition from backstage on to stage was realistic	4.0	4.08	0.90	0.008	4.0	3.67	0.98	0.046
9. The interaction with the audience in the performance space was realistic	3.5	3.42	1.08	ns	3.5	3.42	1.08	ns
10. The spot-lights in the performance space were realistic	4.5	4.08	1.08	0.012	3.5	3.42	1.37	ns
11. The curtains in the performance space were realistic	3.0	3.33	0.98	ns	3.0	3.17	0.93	ns
***SKILL DEVELOPMENT***								
12. The simulation could be used to enhance my musical skills	4.0	4.25	0.86	0.005	4.0	4.25	0.75	0.004
13. The simulation could be used to enhance my technical skills	5.0	4.33	0.98	0.005	4.5	4.33	0.77	0.004
14. The simulation could be used to enhance my communicative/presentational skills	3.0	3.58	1.31	ns	4.0	4.17	0.83	0.006
15. The simulation could be used to help me manage performance anxiety and/or other performance problems	4.0	4.17	0.93	0.007	4.0	4.33	0.77	0.003
16. The simulation could be used to highlight strengths in my performance	4.5	4.33	0.88	0.004	4.0	4.17	0.71	0.004
17. The simulation could be used to highlight weaknesses in my performance	4.5	4.25	0.88	0.006	4.5	4.42	0.66	0.003
18. I would recommend the simulation to people who are interested in developing/refining their performance skills	5.0	4.33	1.10	0.005	4.5	4.42	0.66	0.003
19. I would recommend the simulation to people who are interested in teaching performance skills	5.0	4.42	1.16	0.004	4.5	4.33	0.77	0.004

Ratings for each statement were given from 1 = “strongly disagree” to 5 = “strongly agree.” The significance level p is shown for comparisons of the median rating for each question against a hypothesized median of 3, the scale mid-point, using the Wilcoxon signed-rank test.

#### State anxiety inventory

Immediately prior to each performance, participants were asked to complete Form Y1 (state anxiety) of the State-Trait Anxiety Inventory (STAI; Spielberger et al., 1970). This 20-item questionnaire measures one underlying construct showing the “temporal cross-section in the emotional stream of life of a person, consisting of subjective feelings of tension, apprehension, nervousness, and worry, and activation (arousal) of the autonomic nervous system” (Spielberger and Sydeman, 1994, p. 295). Responses to each question are made on a 4-point scale (1 = “almost never” to 4 = “almost always”), and after inversion of positively worded items, a cumulative score is calculated ranging from low (20) to high (80) state anxiety (for further details, see also Spielberger, 1983).

#### Electrocardiogram

For the subset of 7 participants, ECG data were collected before and during performances in the real and simulated auditions using a wireless Zephyr Bioharness. The device (80 × 40 × 15 mm, weight 35 g) snaps onto an elasticated chest belt (width 50 mm, weight 50 g) and has a sampling rate of 250 Hz. Tests of reliability and validity of the Bioharness have been conducted by Johnstone et al. (2012a,b).

### Procedure

At the start of the study, each of the 11 participants attended a 20-min induction session during which they were informed they would be giving repeated performances of the “Allemande” (with repeats) from J. S. Bach's *Partita No. 2 in D minor* (BWV 1004), a piece all had previously performed in public. Background information on musical experience was collected, and a preliminary health screening was conducted. On explicit questioning, all participants confirmed that they were not currently taking anxiolytic medication or other substances that may affect their perceptions of or physiological responses to performing. For the subset of 7 participants, the Bioharness was fitted and baseline heart rate data were collected. At the end of this session, participants were instructed not to consume alcohol or caffeinated drinks or to smoke for at least 2 h before their forthcoming performances.

#### Performance protocol

For each performance, participants were asked to arrive 20–30 min before their scheduled performance time. They were shown to a “green room” where they were fitted with the Bioharness (where applicable, *n* = 7) and allowed to engage in their usual pre-performance routine (e.g., warming up, practicing, stretching, etc.). Stage calls were given by a member of the research team—acting as the “backstage manager”—at 15 and 5 min before the performance.

At 0 min, each violinist was escorted to the backstage area, asked to complete Form Y1 of the STAI, and required to wait a further 5 min while the backstage manager carried out a scripted check that the stage furniture was correctly placed, the auditorium lights were set, and the audience/audition panel was ready for the performance to begin. During this time, the backstage area was dimly lit, and the participants had sight of the relevant CCTV footage and could hear the low murmur of talking from the audience/audition panel. At the end of these scripted checks, the backstage manager turned to the performer, confirmed that s/he was ready to go on stage, opened the stage door for the musician to walk out, and triggered the applause from the audience (recital) or the greeting from the panel (audition).

While on stage, participants bowed (recital) or returned the greeting (audition) and started their performance as soon as they felt ready. As this was the first experiment using the simulator, polite (but neutral) reactions to the performances were set for the virtual audience and audition panel and no deliberate distractions (coughing, sneezing, talking, phone ringing, etc.) were interjected. Following the end of the performance and shortly into the audience's/panel's response, the stage door opened as a sign to the participant to exit the stage.

For the subset of 7 participants, an additional real audition was organized, in which the same procedure was followed. For parity with the simulated audition, three real panel members (two men, one woman) were shown the footage used in the simulated audition and instructed to provide the exact same neutral response (physically and orally) to all performances.

Performances for all participants were scheduled at the same time on separate days. The order of condition (i.e., simulated recital, simulated audition, real audition) was counterbalanced.

### Data treatment and analysis

#### Simulation evaluation questionnaire

Initial inspection of data from the simulation evaluation questionnaire revealed that 13 of the 19 questions did not meet the criterion for normality (Shapiro Wilk). Therefore, responses to this questionnaire have been analyzed using non-parametric tests (SPSS v19).

#### Electrocardiogram

A key indicator of stress is the temporal fluctuation of the peak-to-peak times in the ECG—the R-to-R (RR) interval—known as heart rate variability (HRV) (Berntson et al., 1997; Berntson and Cacioppo, 2004). This can be studied through time- or frequency-domain analyses. While the time domain can be characterized through a simple calculation of the mean RR or its standard deviation, the frequency domain is studied using power spectral analysis examining HRV's low frequency (LF) and high frequency (HF) components: 0.04–0.15 and 0.15–0.4 Hz, respectively (Berntson and Cacioppo, 2004). The HF element is known to reflect activity of the parasympathetic nervous system (PNS), associated with homeostasis and balance, as well as the respiratory sinus arrhythmia (RSA), naturally occurring variations in heart rate due to breathing. The LF element, although more complex, reflects sympathetic nervous system (SNS) activity, associated with greater arousal. The ratio of LF to HF is widely used as an indicator of the sympatho-vagal balance, with an increase usually reflecting an elevated activation of physical or mental effort (Berntson and Cacioppo, 2004).

In terms of analyses, the mean HRV and its standard deviation are based on the absolute magnitude of the RR interval, whereas in many applications *relative* measures of a process have been shown to exhibit a greater consistency when comparing different individuals, for whom resting heart rate can vary considerably. The relative LF/HF power ratio, although the subject of recent debate as to whether it actually reflects the sympatho-vagal balance (Billman, 2013) and about its accuracy for dynamic stress level assessment (Williamon et al., 2013), has been widely used in the study of stress in performance (Nakahara et al., 2009; for a review, see Billman, 2013).

Here, the ECG data were analyzed using MATLAB (R2013a). Transformation of the RR into a continuous time series with an equivalent sampling frequency of 4 Hz was carried out using cubic spline interpolation as well as a median filter. Bandpass filtering was conducted (0.04–0.4 Hz) via a 4th order Butterworth filter before estimating signal dynamics via overlapping windows using the LF/HF ratio, with LF and HF components obtained via additional 4th order Butterworth filters. In the results below, mean LF/HF ratios are reported as measured at baseline and before and during each performance condition.

## Results

### Performing in the recital and audition simulations

For insight into participants' perceptions and experience of performing in the two simulations, the median rating for each item on the simulation evaluation questionnaire was compared against a hypothesized median of 3, the scale mid-point, using the one sample Wilcoxon signed-rank test (i.e., the non-parametric equivalent of the one-sample *t*-test). As shown in Table 1, the medians for all questions were either 3 or above, and the *p*-values indicate that the medians were significantly higher than 3 for 12 of 19 statements for the recital simulation (63.2%) and 13 of 19 for the audition simulation (68.4%). With median values significantly higher than 3 on the majority of statements, these results suggest that both simulations offered a high quality, realistic performance experience, although with some variation between them.

Focusing more closely on the skill development statements, the participants reported strong potential for both simulations to be used to enhance their own learning and performance skills, to manage performance anxiety and/or other performance problems, and to teach these skills to others. Indeed, 7 of 8 statements for the recital simulation and 8 of 8 for the audition simulation were significantly greater than 3.

In terms of reported anxiety, participants' mean state anxiety score for the recital simulation was 35.09 (*SD* = 11.09) and for the audition simulation was 34.09 (*SD* = 7.09) [according to Spielberger, 1983, moderate levels of anxiety are represented by scores of 36.47 (*SD* = 10.02) for male students and 38.76 (*SD* = 11.95) for female students]. A paired samples *t*-test showed no significant difference between state anxiety scores across the two simulations (*t*_10_ = 0.33, *p* > 0.05), suggesting that the average perceived anxiety before each simulated performance was comparable.

### Performing in the simulated and real auditions

**Figure 4:**
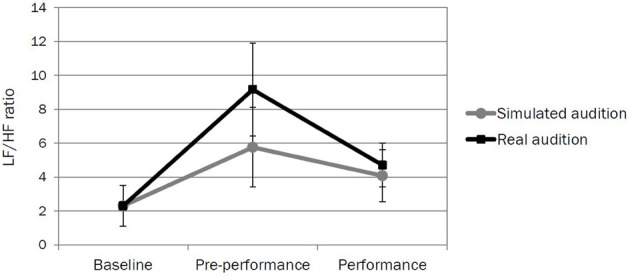
**The mean LF/HF ratio (with *SD*) calculated from ECG data collected for the violinists at baseline, pre-performance, and during performance for the simulated and real auditions**.

For the subset of 7 participants, the LF/HF ratio was calculated from the ECG for (1) the last 5 min of their 20-min induction session (baseline), (2) 5 min immediately before their performance for the simulated and real auditions (pre-performance), and (3) their entire simulated and real auditions (performance). A repeated measures analysis of variance (ANOVA) was run with time of measurement (baseline vs. pre-performance vs. performance) and type of audition (simulated vs. real) as within-subjects variables. The results indicate a significant effect of time [*F*_2, 12_ = 21.01, *p* < 0.001] and audition [*F*_1, 6_ = 9.94, *p* < 0.05] and a significant interaction between time and audition [*F*_2, 12_ = 8.28, *p* < 0.01]. Inspection of Figure 4 suggests that these significant differences are mostly likely due to the high LF/HF ratio in the pre-performance period for the real audition. For levels of self-reported anxiety, participants' mean state anxiety score for the simulated audition was 32.29 (*SD* = 7.39) and for the real audition was 37.43 (*SD* = 7.09), suggesting moderate levels of perceived state anxiety (Spielberger, 1983). A paired samples *t*-test found no significant difference between state anxiety scores across the two auditions (*t*_6_ = −1.65, *p* > 0.05), suggesting that the average perceived anxiety before each audition was comparable.

## Discussion

The aim of this study was to design, test, and explore the possible uses of two new distributed simulation environments for enhancing musicians' learning and performance. These included a small recital and audition setting, both of which offered key visual, auditory, and other environmental cues commonly found backstage and on stage at international performance venues, as well as scripted protocols for how musicians should be guided through them. The violinists found the simulations to be realistic and convincing, with all median ratings for statements in the simulation evaluation questionnaire at the scale mid-point or above (and a majority of statements rated significantly above). In terms of potential uses of the simulations, the musicians were clear in recognizing their positive value for developing performance skills. Moreover, when examining participants' self-reported anxiety and physiological responses to performance stress in the simulated audition versus a real audition, there were no significant differences in state anxiety between the two conditions; while significant differences emerged in physiological responses for the *pre*-performance period, the direction of response (i.e., an increase in the LF/HF ratio) was the same for both, and there were no apparent differences during the performance. Reasons why the higher LF/HF ratio in the pre-performance period did not correspond to higher state anxiety scores could be manifold; musicians may experience the heightened physiological state which accompanies performance as either enabling or debilitating. Nonetheless, in this study comparable psychological and physiological responses were evoked across both auditions, real and simulated.

The distributed simulations used in this research drew upon a selective abstraction of salient procedural and environmental features. In both cases, an important element was the “backstage manager” who guided each musician through the scripted performance process and served as the gatekeeper for their transitions on and off the stage. The recital simulation benefitted particularly from the decor of the backstage area, including signage and lighting, as well as spot-lights in the performance space, while the simulated audition was enhanced especially by auditory cues played in the backstage area.

Unlike previous research in music (Orman, 2003, 2004; Bissonette et al., 2011), which focused on exposure to virtual environments as a means of managing performance anxiety, our results suggest that simulation training may offer wide ranging benefits for musical learning and performance. While further studies are needed to establish these benefits objectively, feedback elicited as part of the simulation evaluation questionnaire suggests that the simulations provided a path by which musicians could not only highlight their performance strengths but also address their weaknesses. As such, they were seen as potentially useful tools for teaching musical skills more efficiently. More generally, this research adds to the growing literature demonstrating that virtual environments can effectively aid learning, especially as they can be accessed repeatedly and consistently, at controlled levels of risk and with pre-defined outcomes. The extent to which the effectiveness of the present simulations will persist through repeated exposure remains to be tested, but findings from other domains would suggest that the prospects of using virtual environments repeatedly and longitudinally to enhance learning and performing are indeed promising (see Rothbaum et al., 2000, 2002).

This study has focused on responses to simulated recital and audition scenarios with relatively neutral and “well behaved” virtual observers. Subsequent studies should examine the influence of disruptions and distractions (e.g., coughing, sneezing, phone ringing, etc.), as well as more positive and negative observer responses. They should also consider recruiting larger samples of musicians, who can specifically be studied in low and high anxiety groups. In addition, there is scope for building and testing further simulated environments: from changing audience size or modifying the number and type of environmental features to involving more performers or altering the performance task itself. It would also be instructive to test whether the degree of background knowledge about the development of the simulations or prior knowledge of whether the performer will encounter a real or virtual audience before they enter the stage (i.e., a blind experiment) would impact how musicians perceive and interact with each simulation.

In general terms, one could argue that distributed simulation training, as employed in this study, has much to offer musicians. However, difficulty arises in mapping out precisely *how* to use simulation in ways that are meaningful to those at different levels of skill, with varying degrees and types of performance exposure, and with very personal experiences of performance anxiety symptoms. Further experimental work that systematically addresses these parameters, carried out using psychophysiological and questionnaire-based measures extended from the current study, could provide such insight.

In this respect, subsequent investigations are needed that explore both generalizable and individual-specific benefits of simulation training. Research should systematically test the effects of simulation on skill acquisition, planning, and self-regulatory strategies, and techniques for improving communication and presentational skills, alongside interventions for managing anxiety. It should also investigate how to match certain types, lengths, and intensities of training to individual musicians' learning and performance objectives. When on stage, it is precisely these objectives that distinguish one performance from another.

## Author contributions

All authors contributed extensively to the work presented in this paper.

### Conflict of interest statement

The authors declare that the research was conducted in the absence of any commercial or financial relationships that could be construed as a potential conflict of interest.

## References

[B1] Axelrod R. (2007). Simulation in the social science, in Handbook of Research on Nature Inspired Computing for Economics and Management, ed Jean-Phillipe R. (Hershey, PA: Idea Group Pub.), 90–100

[B2] Berntson G. G. Bigger J. J. T. Eckberg D. L. Grossman P. Kaufmann P. G. Malik M. (1997). Heart rate variability: origins, methods, and interpretive caveats. Psychophysiology 34, 623–648 10.1111/j.1469-8986.1997.tb02140.x 9401419

[B3] Berntson G. G. Cacioppo J. T. (2004). Heart rate variability: stress and psychiatric conditions, in Dynamic Electrocardiograph, eds Malik M. Camm J. (Hoboken, NJ: Wiley-Blackwell), 56–63

[B4] Billman G. E. (2013). The LF/HF ratio does not accrately measure cardiac sympatho-vagal balance. Front. Physiol. 4:26 10.3389/fphys.2013.00026 23431279 PMC3576706

[B5] Bissonette J. Dube F. Provencher M. D. Moreno Sala M. T. (2011). The effect of virtual training on music performance anxiety, in Proceedings of the International Symposium on Performance Science 2011, eds Williamon A. Edwards D. Bartel L. (Utrecht: European Association of Conservatoires), 585–590

[B6] Clark T. Lisboa T. Williamon A. (in press). An investigation into musicians' thoughts and perceptions during performance. Res. Stud. Music Educ. 16289067

[B7] Ericsson K. A. Krampe R. T. Tesch-Römer C. (1993). The role of deliberate practice in the acquisition of expert performance. Psychol. Rev. 100, 363–406 10.1037/0033-295X.100.3.363

[B8] Gallagher A. G. Ritter E. M. Champion H. Higgins G. Fried M. P. Moses G. (2005). Virtual reality for the operating room: profieciency-based training as a paradigm shift in surgical skills training. Ann. Surg. 241, 364–372 10.1097/01.sla.0000151982.85062.80 15650649 PMC1356924

[B9] Grantcharov T. P. Kristiansen V. B. Bendix J. Bardram L. Rosenberg J. Funch-Jensen P. (2004). Randomized clinical trial of virtual reality simulation for laparoscopic skills training. Br. J. Surg. 91, 146–150 10.1002/bjs.4407 14760660

[B12] Johnstone J. A. Ford P. A. Hughes G. Watson T. Garrett A. T. (2012a). BioHarness multivariable monitoring device. Part I: validity. J. Sports Med. 11, 400–408 24149346 PMC3737934

[B13] Johnstone J. A. Ford P. A. Hughes G. Watson T. Garrett A. T. (2012b). BioHarness multivariable monitoring device. Part II: reliability. J. Sports Med. 11, 409–417 24149347 PMC3737936

[B14] Kassab E. Tun J. K. Arora S. Kind D. Ahmed K. Miskovic D. (2011). Blowing up the barriers in surgical training: exploring and validating the concept of distributed simulation. Ann. Surg. 254, 1059–1065 10.1097/SLA.0b013e318228944a 21738021

[B15] Kenny D. T. (2011). Psychology of Music and Performance Anxiety. Oxford: Oxford University Press 10.1093/acprof:oso/9780199586141.001.0001

[B16] Munz Y. Kumar B. D. Moorthy K. Bann S. Darzi A. (2004). Laparoscopic virtual reality and box trainers. Surg. Endosc. 18, 485–494 10.1007/s00464-003-9043-7 14752633

[B17] Nakahara H. Furuya S. Obata S. Masuko T. Kinoshita H. (2009). Emotion-related changes in heart rate and its variability during performance and perception of music. Ann. N.Y. Acad. Sci. 1169, 359–362 10.1111/j.1749-6632.2009.04788.x 19673808

[B18] Orman E. K. (2003). Effect of virtual reality graded exposure on heart rate and self-reported anxiety levels of performing saxophonists. J. Res. Music Educ. 51, 302–315 10.2307/334565715157123

[B19] Orman E. K. (2004). Effect of virtual reality graded exposure on anxiety levels of performing musicians: a case study. J. Music Ther. 41, 70–78 10.1093/jmt/41.1.70 15157123

[B22] Pertaub D. P. Slater M. Barker C. (2006). An experiment on public speaking anxiety in response to three different types of virtual audience. Presence Teleop. Virt. Environ. 11, 68–78 10.1162/105474602317343668

[B23a] Rothbaum B. O. Hodges L. Smith S. Lee J. H. Price L. (2000). A controlled study of virtual reality exposure therapy for the fear of flying. J. Consult. Clin. Psychol. 68, 1020–1026 10.1037/0022-006X.68.6.1020 11142535

[B23b] Rothbaum B. O. Hodges L. Anderson P. L. Price L. Smith S. (2002). Twelve-month follow-up of virtual reality and standard exposure therapies for the fear of flying. J. Consult. Clin. Psychol. 70, 428–432 10.1037/0022-006X.70.2.428 11952201

[B23] Roy S. (2003). State of the art of virtual reality therapy (VRT) in phobic disorders. Psychnol. J. 2, 176–183

[B24] Satava R. M. (1993). Virtual reality surgical simulator. Surg. Endosc. 7, 203–205 10.1007/BF00594110 8503081

[B25] Slater M. Pertaub D.-P. Barker C. Clark D. M. (2006). An experimental study on fear of public speaking using a virtual environment. Cyberpsychol. Behav. 9, 627–633 10.1089/cpb.2006.9.627 17034333

[B26] Spielberger C. D. (1983). State-Trait Anxiety Inventory STAI (Form Y). Palo Alto, CA: Consulting Psychologists Press, Inc

[B27] Spielberger C. D. Gorsuch R. L. Lushene R. E. (1970). Manual for the State-Trait Anxiety Inventory. Palo Alto, CA: consulting Psychologists Press

[B28] Spielberger C. D. Sydeman S. J. (1994). State-trait anxiety inventory and state-trait anger expression inventory, in The Use of Psychological Testing for Treatment Planning and Outcome Assessment, ed Maruish M. E. (Hillsdale, NJ: Lawrence Erlbaum Associates), 292–321

[B29] Sutherland L. M. Middleton P. F. Anthony A. Hamdorf J. Cregan P. Scott D. (2006). Surgical simulation: a systematic review. Ann. Surg. 243, 291–300 10.1097/01.sla.0000200839.93965.26 16495690 PMC1448942

[B30] Torkington J. Smith S. G. T. Rees B. I. Darzi A. (2001). Skill transfer from virtual reality to a real laparoscopic task. Surg. Endosc. 15, 1076–1079 10.1007/s004640000233 11727073

[B31] Williamon A. (2004). Musical Excellence. Oxford: Oxford University Press 10.1093/acprof:oso/9780198525356.001.0001

[B32] Williamon A. Aufegger L. Wasley D. Looney D. Mandic D. P. (2013). Complexity of physiological responses decreases in high stress musical performance. J. R. Soc. Interface 10, 1–6 10.1098/rsif.2013.0719 24068177 PMC3808549

[B33] Xia J. J. Shevchenko L. Gateno J. Teichgraeber J. F. Taylor T. D. Lasky R. E. (2011). Outcome study of computer-aided surgical simulation in the treatment of patients with craniomaxillofacial deformities. J. Oral Maxillofac. Surg. 69, 2014–2024 10.1016/j.joms.2011.02.018 21684451 PMC3119456

